# Rare copy number variation analysis identifies disease-related variants in atrioventricular septal defect patients

**DOI:** 10.3389/fgene.2023.1075349

**Published:** 2023-02-03

**Authors:** Huan Hu, Zilong Geng, Shasha Zhang, Yuejuan Xu, Qingjie Wang, Sun Chen, Bing Zhang, Kun Sun, Yanan Lu

**Affiliations:** ^1^ Department of Pediatric Cardiology, Xin Hua Hospital, School of Medicine, Shanghai Jiao Tong University, Shanghai, China; ^2^ Key Laboratory of Systems Biomedicine, Shanghai Center for Systems Biomedicine, Shanghai Jiao Tong University, Shanghai, China

**Keywords:** atrioventricular septal defect, congenital heart disease, copy number variation, whole exome sequencing, DYRK1A, OBSCN, TTN

## Abstract

Atrioventricular septal defect (AVSD) is a deleterious subtype of congenital heart diseases (CHD) characterized by atrioventricular canal defect. The pathogenic genetic changes of AVSD remain elusive, particularly for copy number variation (CNV), a large segment variation of the genome, which is one of the major forms of genetic variants resulting in congenital heart diseases. In the present study, we recruited 150 AVSD cases and 100 healthy subjects as controls for whole exome sequencing (WES). We identified total 4255 rare CNVs using exon Hidden Markov model (XHMM) and screened rare CNVs by eliminating common CNVs based on controls and Database of Genomic Variants (DGV). Each patient contained at least 9 CNVs, and the CNV burden was prominently presented in chromosomes 19,22,21&16. Small CNVs (<500 kb) were frequently observed. By leveraging gene-based burden test, we further identified 20 candidate AVSD-risk genes. Among them, *DYRK1A*, *OBSCN* and *TTN* were presented in the core disease network of CHD and highly and dynamically expressed in the heart during the development, which indicated they possessed the high potency to be AVSD-susceptible genes. These findings not only provided a roadmap for finally unveiling the genetic cause of AVSD, but also provided more resources and proofs for clinical genetics.

## Introduction

Atrioventricular septal defect (AVSD), also known as common atrioventricular canal (CAVC) or endocardial cushion defect, is a congenital cardiovascular malformation characterized by defects in the inferior (posterior) atrial septum, the inflow portion of ventricular septum, and the atrioventricular valves (Jacobs et al., 2000). The incidence of AVSD is about 4–5.3/10,000 in live births and about 7% in newborns with congenital heart diseases (Calkoen et al., 2016). According to the atrioventricular valve morphology and the degree of atrial septal defect (ASD), AVSD is classified into three types: partial, transitional and complete (Jacobs et al., 2000). AVSD is associated with genetic syndromes, such as Down syndrome (DS) and heterotaxy syndrome, but also occurs as a simplex trait. Genetic studies indicate that AVSD is genetically heterogeneous, currently over 100 genetic mutations associated with AVSD have been identified, including VEGF-A pathway-related genes (*COL6A1, COL6A2, CRELD1, FBLN2, FRZB*) (Nguyen and Jay, 2014), SHH pathway-related genes (SHH) (Ackerman et al., 2012) and left-right patterning-related genes (*ACVR2B, CFC1, FOXP1, LEFTY2, NODAL, ZIC3*) (Alongi et al., 2020).

Copy number variation (CNV) is gain or loss of genome segments, ranging from hundreds of base-pairs sub-microscopic events to complete chromosomal aneuploidies (Sebat et al., 2004). Usually, CNVs with variation frequency >1% are harmless, and have high copy number change tolerance (Rice et al., 2017; Lye and Purugganan, 2019). In contrast, the CNVs larger than 250 kb tend to cause disease such as developmental disorders (Macé et al., 2018). The pathogenic or rare CNVs lead to disease through increasing expression of dose-sensitive risk genes or altering gene coding or regulatory elements (Lupski and Stankiewicz, 2005; Harel and Lupski, 2017). Recently emerging studies have reported CNVs were closely correlated with CHDs, such as syndromic precocious heart disease (Breckpot et al., 2010), tetralogy of Fallot (TOF) (Greenway et al., 2009), double outlet of the right ventricle (DORV) (Obler et al., 2008), and transposition of the great arteries (TGA) (Costain et al., 2016). CNV in chromosome regions 1q21.1, 2q13, 8q23.1, 16p12.2 and 22q11.2 resulting in abnormal genes expression have been uncovered as important risk factors for cardiovascular developmental abnormalities (Lander and Ware, 2014) (Digilio et al., 2022). Analyzing CNVs and identifying candidate aberrant genes are therefore crucial for prenatal diagnosis and CHDs occurrence assessment.

In this study, we used WES technology and XHMM detection algorithms to analyze the AVSD and control cases and identified 4255 rare CNVs (MAF <0.01) in cohort. Furthermore, 20 genes with rare CNV presented were observed to have significant disease burden in AVSD, and three of them were more likely to be AVSD-causal genes with evident of a high and dynamic expression in the developing heart and hub roles in a CHD core molecular network. Together, this study uncovers a CNV landscape related to AVSD genetics which indicates rare CNVs and their aberrantly modified genes are potentially risk factors for the etiology of AVSD.

## Materials and methods

### Methods

#### Patients’ ascertainment and study populations

The study cohort was obtained from Xinhua Hospital Affiliated to the Shanghai Jiao Tong University School of Medicine from November 2011 to January 2016, including 150 sporadic AVSD patients without familial cases. Patients’ data included sex, age and clinical features (Supplementary Table S1). All patients were diagnosed definitely by echocardiography, cardiac catheterization or surgery. Patients with chromosomal disorders were excluded from the study cohorts. 100 healthy children without developmental abnormalities were randomly selected as controls. All enrollees completed an informed consent form, and the study was approved by the Ethics Committee of Xinhua Hospital.

#### Sample preparation and DNA extraction

2 mL peripheral venous blood was drawn from each patient and healthy control and placed into EDTA anticoagulation tubes, then stored in a −80 °C refrigerator. The genomic DNA was extracted using the QIAamp DNA Blood Mini Kit (Qiagen, Duesseldorf, Germany) following the manufacturer’s instructions. The residual RNA was removed by RNase (Qiagen, Duesseldorf, Germany) at 37 °C for 1 h. The purity of DNA was assessed with a NanoDrop spectrophotymeter (Nanodrop 2000; Thermo Scientific, United States of America).

#### Whole exome sequencing

WES library construction and the sequencing were performed by Biomed lab company (Shanghai, China). In brief, WES libraries were generated by TruSeq DNA Exome Kit according to the manufacturer’s protocol, and the Exome was captured by SureSelect Human All Exon V5 or V6 kit (Agilent Technologies, Inc., United States). Illumina Hiseq (2) and (500) platform was deployed to sequence the shotgun libraries, and paired-end (PE) reads were generated (150 nt for AVSD, 125 nt for control).

#### Reads alignment and BAM file processing

The sequencing reads in FASTQ files of AVSD patients and healthy controls were aligned to the human genome reference (hg19) using BWA-MEM (V.0.7.15). According to the GATK best practice, consequently the BAM files were sorted, and duplicates were marked using Mark Duplicates. Realignment intervals for each BAM file was determined using GATK4 Realigner Target Creator using a list of known indel sites (Mills and 1 kg indels data from the GATK resource bundle ftp://ftp.broadinstitute.org/bundle/hg19/), and base quality recalibration were then performed by Base Recalibrator.

#### CNV calling and rare CNVs identification

XHMM (exon Hidden Markov model) (Fromer et al., 2012) was applied to determine the CNVs in our cases and controls. We run GATK depth of coverage to get sequencing depths, then normalized mean-centered data using PCA (principal component analysis) information, finally used a Hidden Markov Model (HMM) to discover exon-resolution CNV and genotype variation across samples. The parameters were as default (Fromer et al., 2012; Fromer and Purcell, 2014). Furthermore, common CNVs were excluded based on controls and DGV. We removed CNVs of overlapping rate with common CNVs larger than 50% (Liu et al., 2018).

#### Candidate genes screening

Gene-based burden test was conducted on the genes affected by rare CNVs to identify AVSD-associated genes. Fisher-exact test were used for the frequency comparisons between AVSD cohort and control, and *p*-value less than 0.05 were considered to be statistically significant. The genes with *p* < 0.05 were candidate genes.

#### Functional enrichment analysis and network analysis

Functional annotation was performed in DAVID (http://david.abcc.ncifcrf. gov/) for Gene ontology (GO) terms analyses. The network analysis between candidate genes and known genes related to AVSD (http://chdgene.victorchang.edu.au/) was performed by STRING (https://www.string-db.org/). Cytoscape and its plugin cytoHubba were deployed to detect the hub nodes genes and visualize the PPI networking.

#### Bulk RNA-seq and single-cell analysis

The expression matrix of bulk RNA-Seqs of heart from 4 wpc (weeks post-conception) to 19 wpc was obtained from the Cardoso-Moreira M et al. (Cardoso-Moreira et al., 2019) study, and the expression was quantified as CPM (counts per million). Single-cell RNA-Seq for the human embryonic heart (5 wpc∼24 wpc) was achieved from the study of Yueli Cui et al. (Cui et al., 2019) (GSE106118). Gene expression was quantified as CPM, reflecting the normalized number of unique molecular identifier (UMI) sequences. Cell filtration was carried out as recommended (Cui et al., 2019). The Seurat R package (Stuart et al., 2018) was used for the following analysis.

#### Statistical analysis

Statistical analysis of the data was completed by GraphPad Prism, version 8.4 (GraphPad Software, San Diego, California, United States of America). Two-sides *p* < 0.05 was considered as statistical significance. A Spearman’s correlation test was used to investigate the correlation between the chromosome length and CNV counts.

## Results

### Clinical characteristics of patients

A total of 150 AVSD patients including 76 males (50.7%) and 74 females (49.3%) were recruited in the study. Clinical diagnosis depends on the type of AVSD associated with degree of shunting. Complete AVSD can be diagnosed at early period, while partial AVSD can remain asymptomatic for years (Calkoen et al., 2016). In our study, 64 patients (42.7%)were infants less than 1 year old, 60 patients (40.0%) were between 1 and 3 years old, and only 26 patients (17.3%) were more than 3 years old (Table 1). 125 (83.3%) patients had complete AVSD, 22 (14.7%) had partial AVSD (*n* = 22) and 3 (2.0%) had transitional AVSD. In addition to AVSD as the primary diagnosis, some other cardiac malformations were recorded, including patent ductus arteriosus (PDA) (10.7%, *n* = 16), single atrium or single ventricle (20.0%, *n* = 30), heterotaxy (22.0%, *n* = 33), pulmonary stenosis or pulmonary atresia (21.3%, *n* = 32) and conotrunical defect (11.3%, *n* = 17). . More than half of the patients suffered complications (55.3%, *n* = 83) such as cardiac insufficiency, heart failure, pulmonary hypertension and pericardial effusion (Table 2). The detailed clinical features were presented in Supplementary Table S1.

**TABLE 1:** **Demographic characteristics of patients**.

	Number of patients (%)
Sex	
male	76 (50.7%)
female	74 (49.3%)
Age	
<1 year	64 (42.7%)
1∼3 years	60 (40.0%)
>3 years	26 (17.3%)

**TABLE 2:** Diagnosis of the patients with AVSD.

	Number of patients (%)
Phenotype	
Partial	22 (14.7%)
Intermediate	3 (2.0%)
Complete	125 (83.3%)
Other cardiovascular malformations	
Patent ductus arteriosus	16 (10.7%)
Single atrium or single ventricle	30 (20.0%)
Heterotaxy	33 (22.0%)
Pulmonary stenosis or pulmonary atresia	32 (21.3%)
^a^Conotrunical defect	17 (11.3%)
Complications	37 (24.7%)
Yes	83 (55.3%)
No	39 (44.7%)

^a^ Conotrunical defect includes persistent truncus arteriosus, transposition of the great arteries and double outlet right ventricle.

^b,^Complications includes cardiac insufficiency, heart failure, pericardial effusion pulmonary hypertension.

### The landscape of rare CNVs in AVSD

**FIGURE 1:**
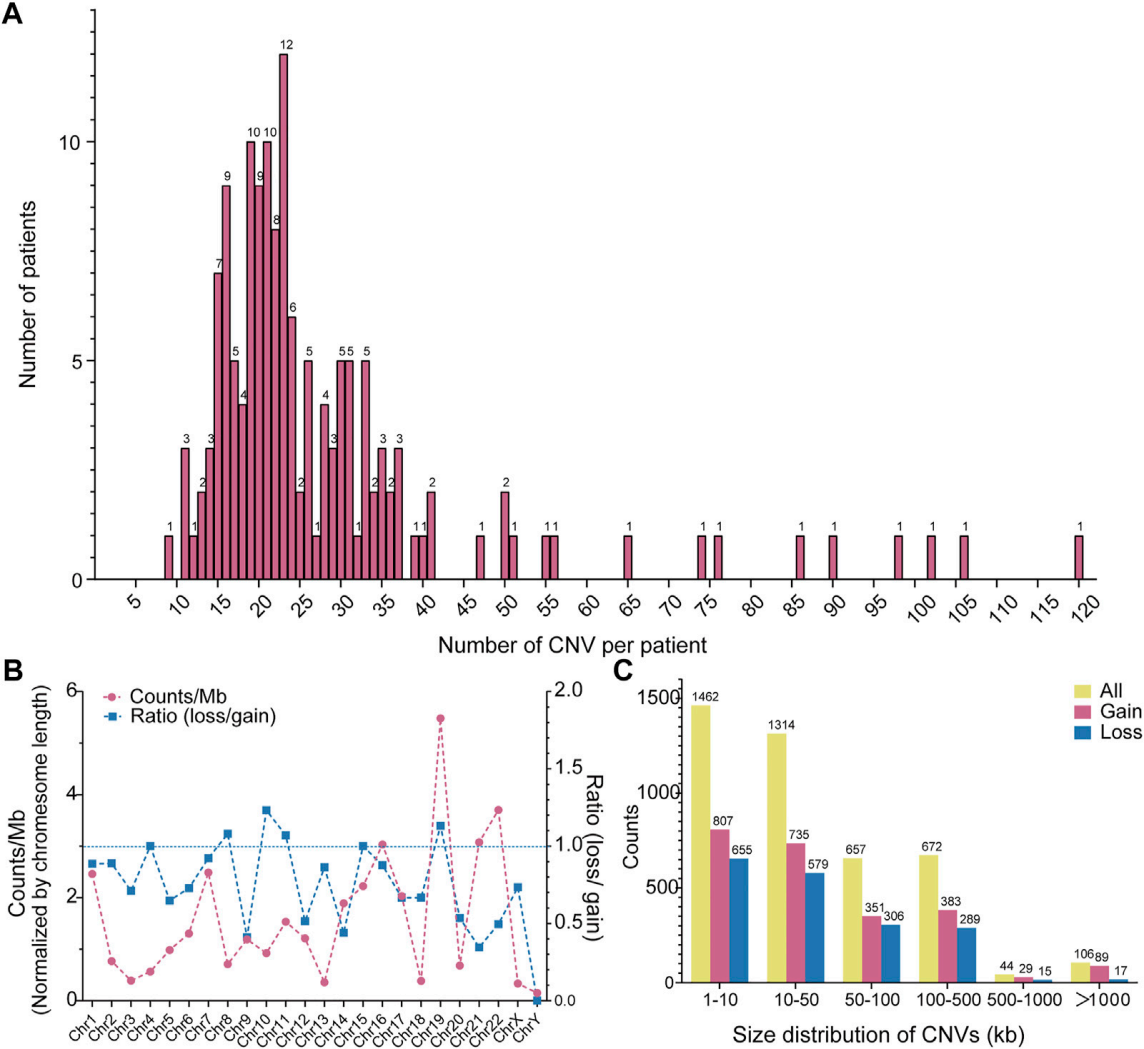
Distribution of CNVs in AVSD. **(A)** Frequency distribution of CNV counts in each individual. The *Y*-axis represents the number of patient and the *X*-axis represents CNV count per individual. **(B)** Chromosomes distribution of CNVs and the ratio of deletion (loss) and duplication (gain). The red line represents the CNV count of each chromosome, the blue line represents the ratio of loss (deletion) and gain (duplication). **(C)** Size distribution of CNVs. The yellow, red and blue column indicate all, gain and loss CNV count, respectively. CNV, copy number variation; AVSD, atrioventricular septal defect.

As aforementioned, CNV is one of the fundamental chromosomal aberrations for CHD. We examined across cohorts. Whole exons of patients and controls were sequenced by the illumina sequencing platform and displayed similar sequencing metrics on target regions. Percentage of Sequencing quality score of each base lager than Q20 is 96.24% across all samples. Mean read mapping quality score is 46.27. Mean coverage depth on target region is 114.51. Genome-wide detection of CNVs was performed by XHMM through leveraging the large-scale nature of sequencing projects to discern patterns of read-depth biases (Fromer et al., 2012). The classification of CNV types, duplication or deletion, was based on the sequencing depth of exons. In our study the CNVs of overlapping rate larger than 50% with CNVs reported on DGV and controls were categorized into common CNVs (Liu et al., 2018), the rest CNVs were defined as rare CNVs. The pathogenic CNVs were those have been previously reported in the available public database. Finally filtered rare CNVs were annotated for gene content and frequency. We identified total 4255 rare CNVs in our 150 AVSD cohort, including 2394 genetic duplications and 1861 genetic deletions (Supplementary Table S2). The numbers of rare CNVs in each patient were ranged from 9 to 120, and the range of 11–37 CNVs per person was mainly distributed (86.67%, *n* = 130). 15 patients have more than 45 CNVs per person, and the maximum number is 120 (Figure 1A).

Further, to explore the CNV characteristics in our study, we partitioned the data across chromosome and range of CNV size. The correlation between chromosome length and CNV counts is not statistically significant according to the spearman rank correlation coefficients (rho) test (*r* = 0.1643, *p* = 0.4428) (Supplementary Figure S1), suggesting a predilection for the chromosome occurrence of CNV in AVSD patients. We normalized the number of rare CNVs by their located chromosome length, and showed a CNV chromosomal enrichment existed in Chr19, 22, 21 and 16, especially in Chr19. In contrast, they were less enriched in Chr3, 18,13 and X. There was minimum number in ChrY. Most of chromosomes possessed more gain-than loss-type of CNVs, especially chr14, 21, 22 and Y. The loss and gain CNVs were similar in Chr 4, 7, 8, 10, 11, 15&19 (Figure 1B). The length of the rare CNVs identified ranged from 97bp to 21 Mbp with a median of 26.1 Kbp (Supplementary Table S2). We divided rare CNVs into six groups based on CNV size (Figure 1C). There was a bias towards small CNVs (<500 kb), and maximum number existed in the range of 0–10 kb (34.3%, *n* = 1,462). However, large CNVs (>500 kb) also existed but only occupied 2.87% (*n* = 122). The counts of genetic duplication were higher than genetic deletions in all size groups.

**FIGURE 2:**
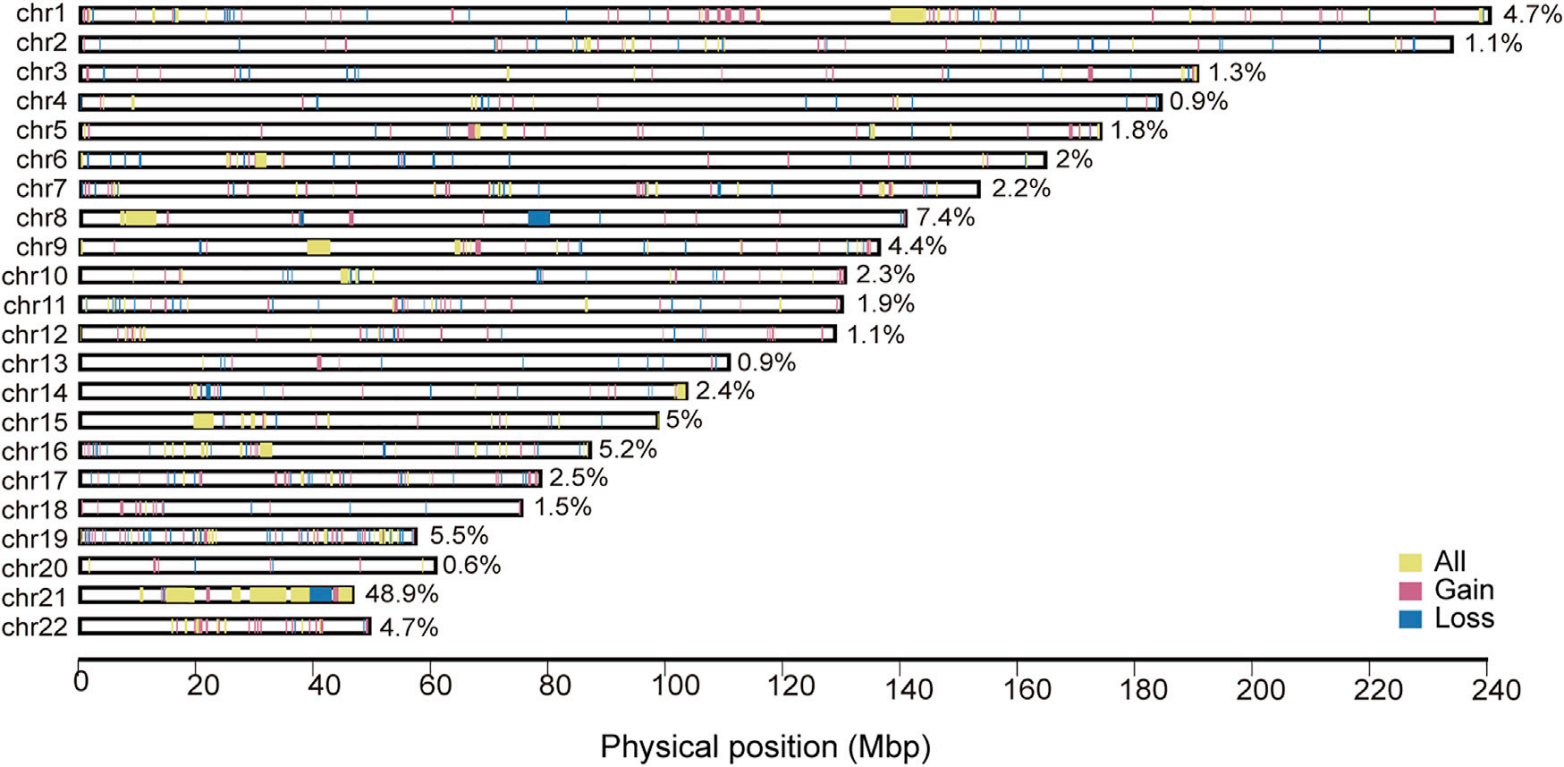
Genomic distribution of CNVRs of AVSD. The red denote CNVRs associated with duplications, the blue denote CNVRs associated with deletions, the yellow denote CNVRs associated with both types. The width of color block represents the size of each CNVR. CNVR, copy number variation region.

We obtained unique CNV regions (CNVRs) by aggregating the overlapping CNVs (with at least 1bp of overlap) that were identified across all of the case samples. . The genome-wide chromosomal map of CNVRs in autosomes were shown in Figure 2. The proportion of chromosome covered by CNVRs varied vastly between chromosomes, ranging from 0.6% of Chr20 to 48.9% of Chr21. The big proportion of CNVR on chr21 may indicates some patients may have chromosomal aneuploidy such as Down’s syndrome even they were not diagnosed when enrolled. It also suggested that CNV on the trisomic chromosome 21 could increase the risk for AVSD (Sailani et al., 2013). Large CNVRs mainly locate in Chr1,8,9 and 21. In addition, most of CNVRs were composed of gain-type CNVs rather than loss-types, but many CNVRs also includes both.

### Identification of known pathogenic CNVs

It is well established that certain recurrent CNVs were associated with CHDs. To examine the possibility that known pathogenic CNVs were associated with AVSD in our cohort, we compiled the published pathogenic CNVs from DECIPHER (http://decipher.sanger.ac.uk/) and ClinGen database (https://www.clinicalgenome.org/data-sharing/clinvar/). We detected 4 CNVRs overlapped at least 50% length with previously pathogenic CNVRs in our 7 AVSD patients, including 1q21.1, 2q13, 8p23.1 and 16p12.2. These CNVs have been reported to associate with a range of cardiac defects in DECIPHER, ClinGen database and published literatures, including coarctation of the aorta (CoA), interrupted aortic arch (IAA) (Christiansen et al., 2004), left ventricular outflow tract obstructive defects (LVOTO) and ventricular septal defect (VSD) (Lander and Ware, 2014), ASD, AVSD, pulmonary stenosis (PS) (Barber et al., 2007), laterality defects, cardiomyopathies (Digilio et al., 2022), hypoplastic left heart and TOF(Girirajan et al., 2010). In our study, one patient (No. 191252) was diagnosed as complete AVSD with 1.2 Mb 1q21.1 deletion encompassing 16 genes including known CHD risk genes *APC6, BCL9, CHD1L, FM O 5, PRKAB2* and *GJA5* (Tomita-Mitchell et al., 2012). Three patients (No.223503, No.224312 and No.61) harbored 2q13 deletion with the length from 107kb to 135 kb containing *MALL* and *NPHP1*, and all of them suffered the complete AVSD. One patient (No. 183217) was diagnosed as partial AVSD with 2.4 Mb 8p23.1 deletion consisted of 19 genes including a well-known CHD-related gene *GATA4, SOX7* (Tomita-Mitchell et al., 2012). Two patients (No. W101 and No.172905) with a 298kb–438 kb deletion in 16p12.2 (10 genes within this region and none of them were defined as CHD disease genes) were diagnosed as complete AVSD (Table 3). These results together indicated the pathogenic potentials of the CNVs we identified.

**TABLE 3:** Pathogenic CNVs.

Sample name	Diagnosis	^#^Chr	Start	End	Region size	cytoBand	CNV type
191252	AVSD (complete)/PH	chr1	146584283	147806803	1222520	1q21.1-q21.2	DEL
223503	AVSD (complete)/LSVC/MR	chr2	110855124	110962791	107667	2q13	DEL
224312	AVSD (complete)	chr2	110855124	110962647	107523	2q13	DEL
61	AVSD (complete)/HTX/PD/SV/Asplenia syndrome	chr2	110827494	110962635	135141	2q13	DEL
183217	AVSD (partial)/AVR/LSVC/PH	chr8	8098153	10588092	2489939	8p23.1	DEL
W101	AVSD (complete)/HTX/DORV/PS/PDA	chr16	21391473	21830024	438551	16p12.2	DEL
172905	AVSD (complete)/PDA/PH	chr16	21608536	21907102	298566	16p12.2	DEL
207063	AVSD (partial)	chr21	26946247	48084365	21138118	21q22.11-21q22.3	DUP
210973	AVSD (complete)/PH/MR	chr21	26946247	48084365	21138118	21q22.11-21q22.3	DUP
209182	AVSD (complete)/PH	chr21	26946247	45837963	18891716	21q22.11-21q22.3	DUP
221308	AVSD (complete)/PH	chr21	30250542	48084365	17833823	21q22.11-21q22.3	DUP
241802	AVSD (partial)/MR/TR/PH	chr21	30250542	48084365	17833823	21q22.11-21q22.3	DUP
201013	AVSD (complete)/LSVC/PH	chr21	30251994	48084365	17832371	21q22.11-21q22.3	DUP
225462	AVSD (complete)/PH	chr21	30251994	48084365	17832371	21q22.11-21q22.3	DUP
264282	AVSD (complete)/AVR/TR	chr21	30251994	40551975	10299981	21q22.11-21q22.2	DUP
208738	AVSD (complete)/PDA/LSVC/PH	chr21	30257471	48084365	17826894	21q22.11-21q22.3	DUP
204113	AVSD (complete)/PS/PH	chr21	30303475	40695048	10391573	21q22.11-21q22.2	DUP
205219	AVSD (complete)/PH	chr21	34117827	48084365	13966538	21q22.11-21q22.3	DUP
172905	AVSD (complete)/PDA/PH	chr21	37408332	48084365	10676033	21q22.13-21q22.3	DUP

AVR, atrioventricular valve reflux; AVSD, atrioventricular septal defect; DORV, double outlet right ventricle; HTX, heterotaxy; LSVC, left superior vena cava; MR, mitral regurgitation; PD, pulmonary dysplasia; PDA, patent ductus arteriosus; PH, pulmonary hypertension; PS, pulmonary stenosis; SV, single ventricle; TR, tricuspid regurgitation.

Although we removed the clinical diagnosis of chromosomal aneuploidy, the enrichment of CNVs on chromosome 21 (Supplementary Table S2) reminded us of trisomy 21. It is well known that not all human chromosome 21 loci are required for the manifestation of DS (Pl et al., 1961). The region on distal 21q22.13 of only 34 kb (interval from 37929229 to 37963130, genome version: GRCh38/hg38) has been identified as a highly restricted DS critical region for the phenotype, whose duplication is shared by all DS subjects and is absent in all non-DS subjects (Pelleri et al., 2016). 12 subjects in our cohort contained large segment duplications of 10Mb–21 Mb on Chr21, spanning the critical region 21q22.13 (Table 3). It indicated that these patients may have partial DS or DS but never been diagnosed before. These large duplication variants contained at least 265 genes, including genes (*BACH1, SOD1, CRYZL1, ATP5O, C21orf2*) with role in energy and reactive oxygen species metabolism, genes (*SIM2, DYRK1A, GART, PCP4, S100B*) with role in brain development and genes (*N6AMT1, SLC19A1, DNMT3L, CBS, FTCD*) with role in folate and methyl group metabolism (Roizen and Patterson, 2003). Overexpression of these genes may lead to various phenotypes of Down’s syndrome such as craniofacial and cardiac maldevelopment.

### Establishment of the gene-disease relationship with burden test

**FIGURE 3:**
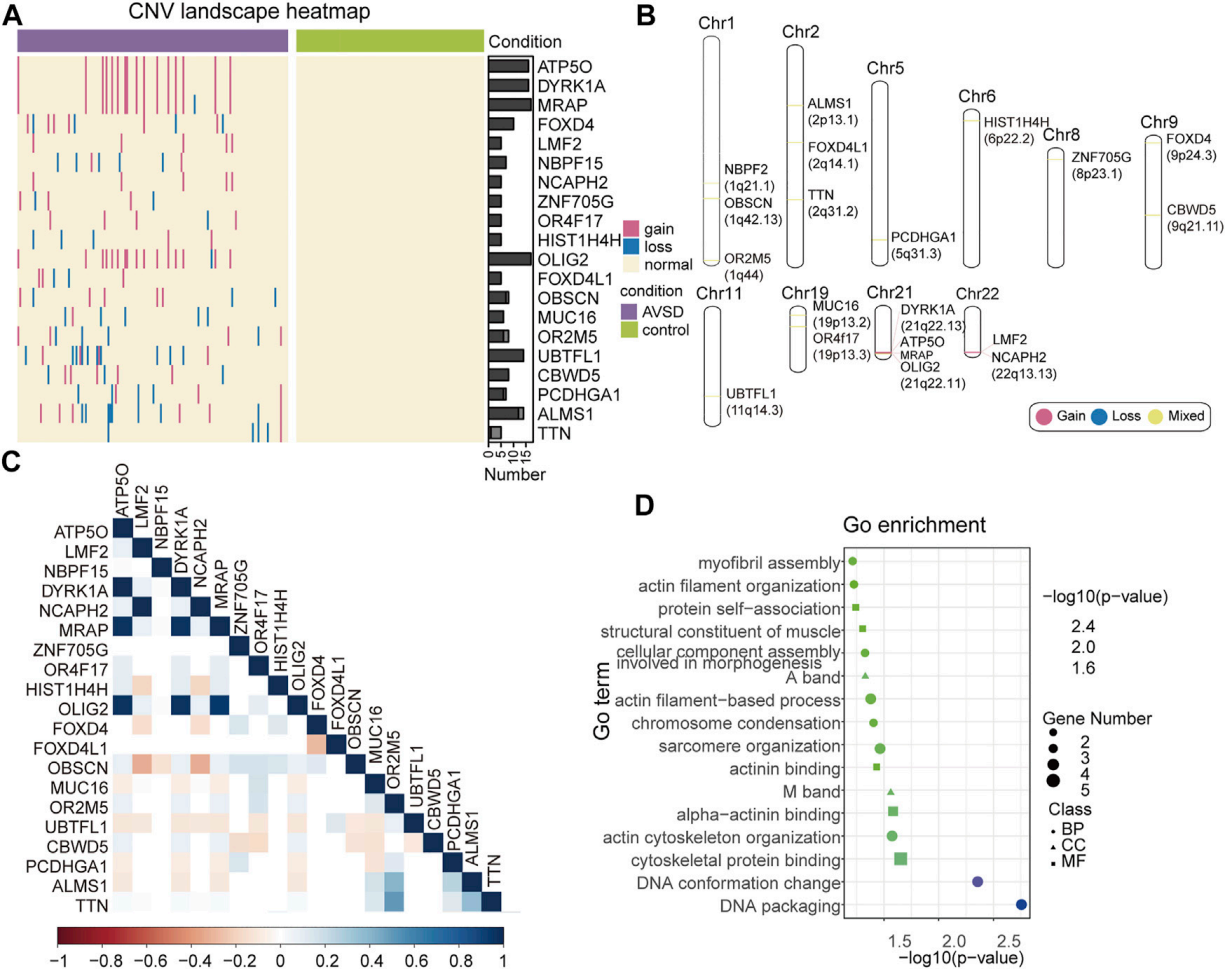
The landscape of candidate gene. **(A)** Heatmap of 20 candidate genes expression. The red line represents gene duplication, the blue line represents gene deletion, the normal subjects are depicted in yellow. **(B)** Candidate genes in the chromosomes. Gene deletions and duplications are shown in blue and red, genes with both variation types are show in yellow. **(C)** Correlation heatmap of candidate gene. The color bar indicates the corresponding R-value. **(D)** GO analysis of candidate genes. GO enrichment analysis in DAVID includes biological processes (BP), cell component (CC), and molecular function (MF). The *x*-axis shows the -log10 *p*-value. The number of candidate genes annotated with a GO term is mapped to the scatter plot by point size.

Although the pathogenesis of a few of identified CNVs were defined, the most part of them remain unclear. To establish the link of these CNVs to AVSD and identify the novel risk genes, gene-based burden test was deployed to determine the candidate genes. As shown, 20 candidate genes associated with 182 CNVs fragments were significantly enriched in the AVSD (*p* < 0.05) (Figure 3A; Supplementary Table S3), and the CNVs within none of them were observed in control cohort. These genes were distributed across 10 chromosomes and Chr21 had the most (Figure 3B).

Notably, the gain variants of four candidate genes (*ATP5O, DYRK1A, MRAP* and *OLIG2*) occurred in the same patients (10.67%, *n* = 16) and all located on 21q22. Similar to the aforementioned genes in 21q22, the gain variants of *LMF2* and *NCAPH2* that located on 22q13.13 occurred in same patients as well (3.3%, *n* = 5) (Figure 3B), which indicated that these genes were inherited in the same linkage (Figure 3C). The rest of genes were associated with both loss- and gain-type of CNVs indicating either dose-sensitivity or functional aberration of these genes may be attributable to AVSD pathogenesis. So far, none of them had been reported to link to heart developmental defects. To further elucidate the function of these candidate genes, we further performed Go term analysis. The result revealed that their functions were related to myofibril assembly, actin filament organization, chromosome condensation and sarcomere organization, etc (Figure 3D), which play critical roles in the heart development.

### The association of candidate genes with heart development

**FIGURE 4:**
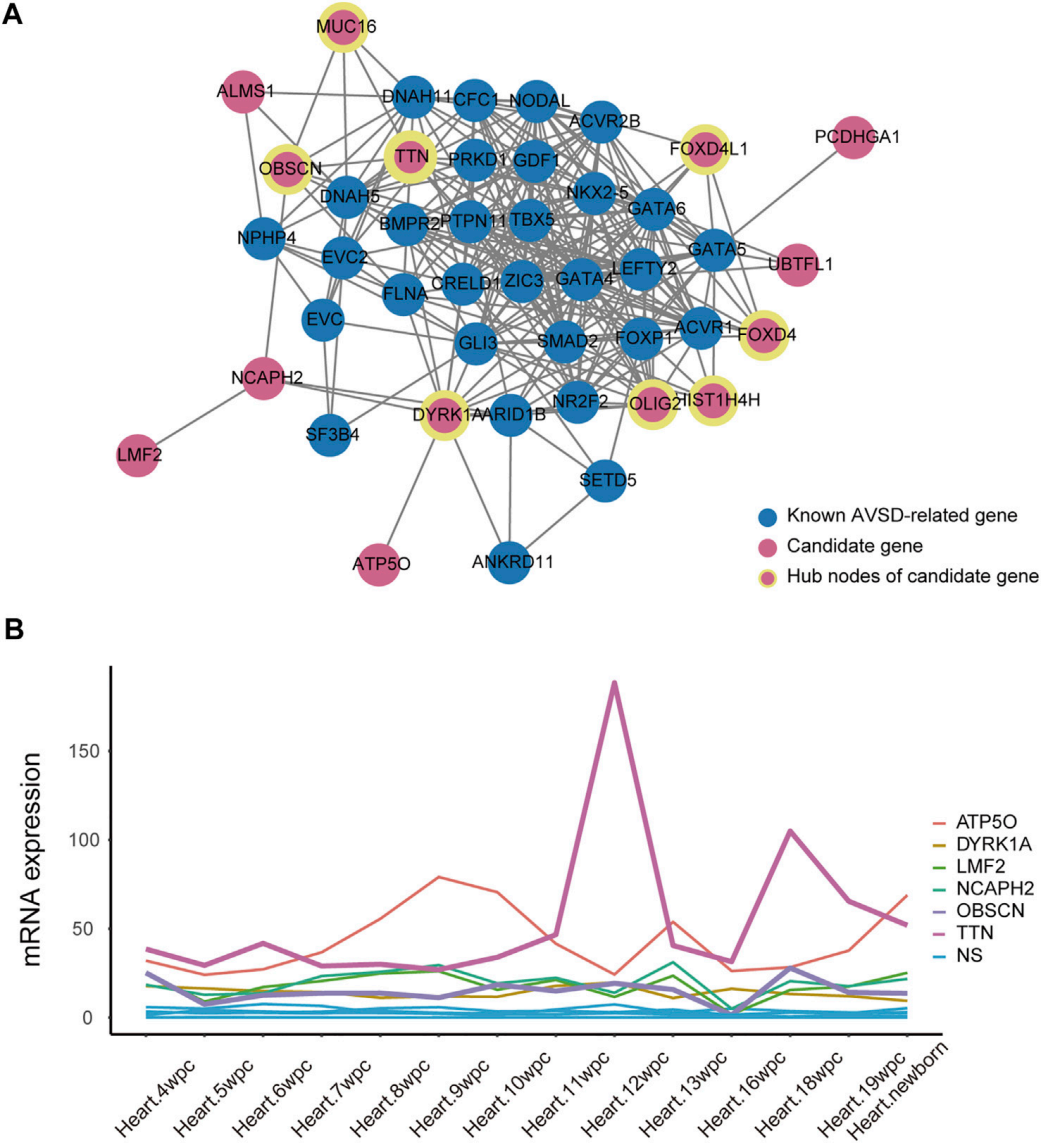
The association between candidate gene and heart development. **(A)** Protein-protein interactions analysis of 31 known AVSD-related genes (shown in blue) and 20 candidate genes (shown in red) using STRING website tools. The hubba nodes of candidate genes are shown in red circle with yellow edge. The remaining proteins without significant interaction with this main network were not shown. **(B)** The quantitative expression of candidate genes during embryonic development from 4 to 19 weeks. AVSD, atrioventricular septal defect.

To further validate the role of candidate genes in heart development, we performed protein-protein interaction analysis of 31 known AVSD-related genes and 20 candidate genes with STRING, and further deployed cytoHubba to detect the hub nodes The intensive and complex interactions were observed between these genes (Supplementary Table S4). Among 20 candidate genes, *OLIG2, DYRK1A, TTN, FOXD4, FOXD4L1, HIST1H4H, OBSCN* and *MUC16* ranked on the top of the maximal clique centrality (MCC) score list (Figure 4A; Supplementary Table S6), and possessed the features of hub nodes. The CHD-related genes are usually expressed in a high and dynamic level in the process of heart development. We therefore analyzed the published human embryonic heart bulk RNA-seq dataset to investigate the temporal expression of 20 candidate genes. Only six candidate genes (*ATP5O, DYRK1A, LMF2, NCAPH2, OBSCN* and *TTN*) exhibits high and dynamic expression from 4 weeks to 19 weeks in human embryonic heart (Figure 4B). Integrating the results of protein interactions and genes temporal expression suggested that the *DYRK1A, OBSCN* and *TTN* gene may play important roles in cardiac development.

### Potential risk genes for AVSD

**FIGURE 5:**
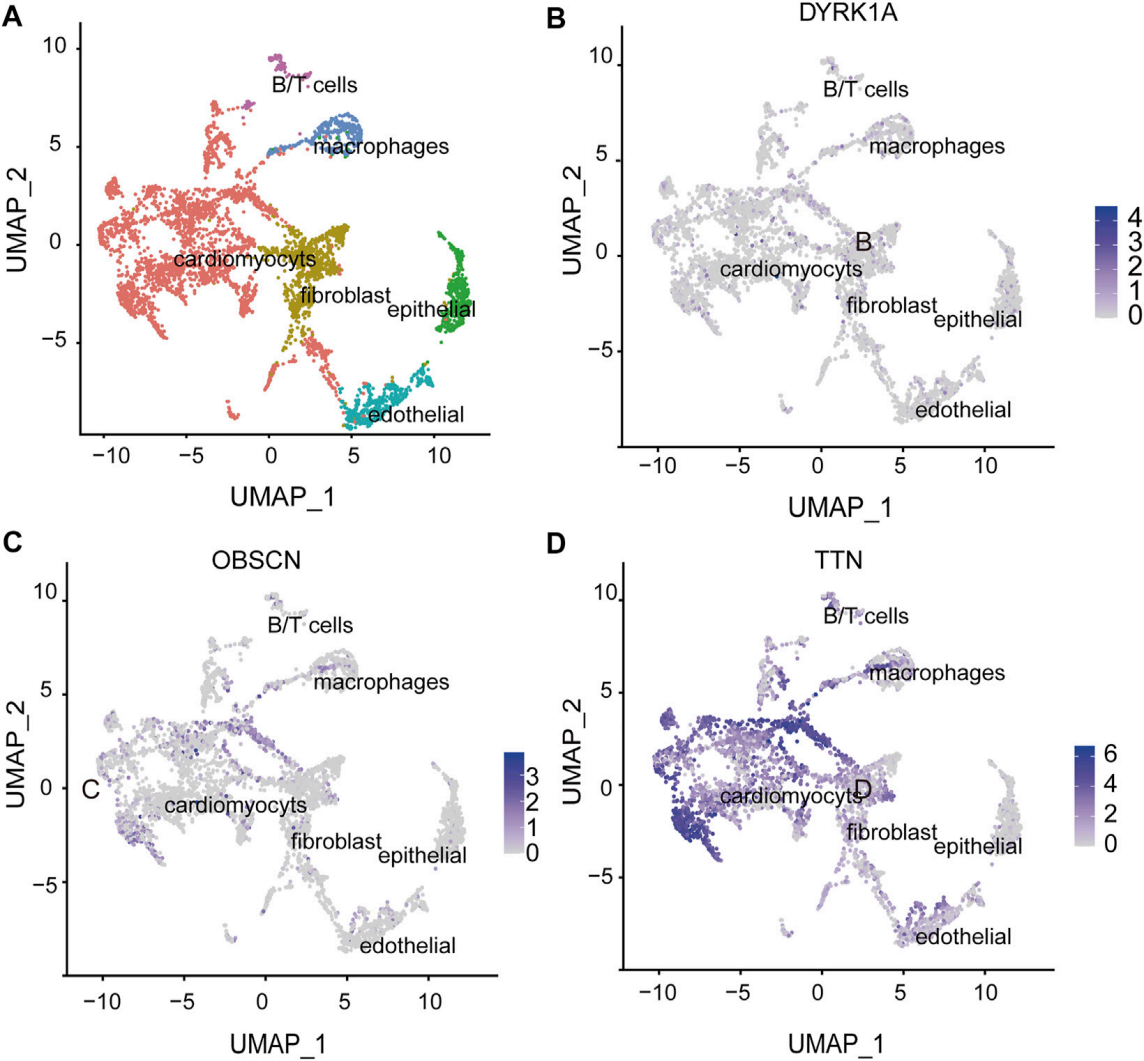
The spatial expression of potential risk genes in the heart. **(A)** Single-cell atlas of embryonic hearts cells. Uniform manifold approximation and projection (UMAP) plot illustrate six clusters of human embryonic hearts cells. **(B–D)** The spatial expression of DYRK1A, OBSCN and TTN in the heart cell clusters. The intensity of the blue point represents the extent of genes expression. DYRK1A, dual-specificity tyrosine-(Y)-phosphorylation regulated kinase 1A; OBSCN, Obscurin; TTN, Titin.

We defined *DYRK1A, OBSCN* and *TTN* as potential risk genes by the foregoing gene expression and function analysis. We further classified cardiac cells into six populations based on a single-cell sequencing data (Cui et al., 2019) (Figure 5A) and analyzed the expression of potential risk genes in different cell types of the heart. *DYRK1A* (dual-specificity tyrosine-(Y)-phosphorylation regulated kinase 1A) is phosphorylation kinase that can phosphorylate target substrates at Ser or Thr residue and is associated with multiple pathways (Park et al., 2009). The *DYRK1A* expression during heart development was relatively stable (Figure 4B), and *DYRK1A* was moderately expressed in cardiomyocytes, endothelial cells and fibroblasts in the human embryonic heart (Figure 5B). As a hub node, it was connected to 11 AVSD-related proteins in the protein-protein interaction network (Figure 4A). *DYRK1A* gene has been previously confirmed to play a role in the cardiomyocyte proliferation by regulating cell cycle (Hille et al., 2016). The recent research has found that *DYRK1B* plays an important role in mitochondrial bioenergetics and the progression of cardiac hypertrophy and heart failure (Zhuang et al., 2022).

*OBSCN* (Obscurin) is a giant sarcomeric protein that play essential roles in myofibrillogenesis, cytoskeletal organization, and Ca2+ homeostasis (Ackermann et al., 2014). *OBSCN* located at 1q42.13 locus, and there are eight subjects with *OBSCN* variation (*p* = 0.005). Three of them were loss of function and five were gain of function (Supplementary Table S5). There was an expression peak of *OBSCN* in 16 wpc and one expression trough in 13wpc during human heart development (Figure 4B). *OBSCN* was moderately expressed in the human embryonic heart, and relatively high expression in cardiomyocytes (Figure 5C). It was associated with 4 AVSD-related proteins in the protein-protein interaction network as a hub node (Figure 4A). Several clinical studies previously reported that *OBSCN* missense variants or mutations are associated with left ventricular non-compaction (LVNC) (Rowland et al., 2016), hypertrophic myocardiopathy (HCM) (Arimura et al., 2007) and dilated cardiomyopathy (DCM) (Marston et al., 2015).

*TTN* (Titin) is also a large sarcomeric filament spanning from Z disk to M-band, and important for myocardial passive stiffness and stress sensitive signaling (Granzier and Irving, 1995; Cazorla et al., 2001). TTN located at 2q31.2 locus, and there are six subjects with *TTN* variation (*p* = 0.03). Four of them were loss of function and two were gain of function (Supplementary Table S5). There were two expression peaks of *TTN* in 11 wpc and 16 wpc during human heart development (Figure 4B). *TTN* were most abundantly and widely expressed in the embryonic heart, and especially in the cardiomyocytes (Figure 5D). Titin has three variable splicing isoforms: adult N2BA, adult N2B, and fetal cardiac Titin. The isoforms ratio determinate Titin-based passive tension and diastolic filling during development (LeWinter and Granzier, 2014). *TTN*, as a hub node, was linked to 11 AVSD-related proteins in the protein-protein interaction network (Figure 4A). It is also an important cardiomyopathy gene, and its mutation is associated with dilated cardiomyopathy (Herman et al., 2012) and arrhythmogenic right ventricular dysplasia (Taylor et al., 2011).

## Discussion

The study demonstrated 4255 rare CNVs landscape in 150 AVSD cases and describes the distribution characteristics of the rare CNVs in chromosome, rare CNVs length and individual frequencies. This suggests that rare loss and gain variations play significant roles in the pathogenesis of AVSD. We also screened 20 significant candidate genes by gene burden, and identified that *DYRK1A, OBSCN* and *TTN* are potential risk genes of AVSD.

AVSD can occurs as a simplex trait, but also is associated with genetic syndromes such as Noonan syndrome, Holt-Oram syndrome and especially Down’s syndrome. Previous studies found that AVSD were the most common CHDs in Down’s syndrome, the incidence was from 9.6% to 46.4% (Mourato et al., 2014; Epçaçan et al., 2019). However, it remained ambiguous whether Down’s syndrome was genetically linked with AVSD (Sailani et al., 2013). Here, we unbiasedly found Chr21 were enriched with CNVs especially large size of CNVs, and 12 subjects may have partial DS or DS but never been diagnosed before by CNVs analysis. These data suggest that Down’s syndrome is genetically associated with AVSD. Further sequencing approaches for chromosomal abnormality such as CGH (comparative genomic hybridization), FISH (fluorescence *in situ* hybridization) or karyotype were required for validation this hypothesis. Moreover, the causal gene for AVSD in Down’s syndrome is largely unknown. Our study found *ATP5O, DYRK1A, MRAP* and *OLIG2* have high burden in AVSD, especially *DYRK1A* that is highly and dynamically expressed in the developing heart, and the gain of function rather loss of function of it will leads to AVSD and Down’s syndrome phenotype. We hypothesized that CNVs on specific regions of chromosome 21 might constitute an additional genetic risk factor for AVSD.

Non-syndromic AVSD patients always have associated cardiac anomalies, including TOF, LVOTO, PDA, DORV, unroofed coronary sinus syndrome et al. (Hoohenkerk et al., 2010; Jegatheeswaran et al., 2010; Atz et al., 2011; Irving and Chaudhari, 2011). In our study, 16 patients contained the DYRK1A variant, all but one had complete AVSD, four (25%) of which also had a diagnosis of patent ductus arteriosus (PDA). 7 patients contained the OBSCN variant, two had partial AVSD, five had complete AVSD, and 3 patients (42.86%) of which also had left superior vena cava (LSVC). Those indicate the complexity of the AVSD associated phenotypes. 6 patients had the TTN variant, all with complete AVSD. And they all had comorbid cardiac malformations, five (83.33%) with heterotaxy (Right atrial isomerism), four (66.67%) with PS/PA, three (50%) with SA/SV, and two (33.33%) with DORV. This suggests that TTN is a key gene in cardiac development and its expression change can result in severe and complex cardiac phenotypes.

Four pathogenic CNVs identified in our study were validated to be pathological, including 1q21.1, 2q13, 8p23.1 and 16p12.2. 1q21.1 recurrent microdeletion has reported in severe developmental delay and multiple congenital anomalies (Brunetti-Pierri et al., 2008; Mefford et al., 2008). Previous studies strongly suggest that *GJA5*, encoding a protein forming gap junctions, is the critical gene for the CHD phenotype in this locus (Gu et al., 2003; Soemedi et al., 2011). 2q13 recurrent microdeletion are associated with developmental delay, autism spectrum disorder, attention deficit hyperactivity disorder, craniofacial malformation, CHD and other features. FBLN7 and *TMEM87B* in 2q13 locus could confer susceptibility to congenital heart defects (Russell et al., 2014). However not *FBLN7* and *TMEM87B* but *MALL* and *NPHP1* in 2q13 locus were found in our data. The association between the two genes with CHD phenotype required further research. 8p23 recurrent microdeletion encompassing *GATA4* has previously been associated with multiple malformations that include CHD (Pehlivan et al., 1999). *GATA4* is one of the most fundamental transcription factors controlling heat lineage commitment and chamber formation (Wat et al., 2009). Deletion of *GATA4* in mice results in cardiac developmental abnormality and early lethality by E9.5 (Molkentin et al., 1997). 16p12.2 recurrent microdeletion have been reported in association with non-syndromic deafness (Shahin et al., 2009), developmental delay and cardiac malformations (Girirajan et al., 2010). The recurrent detection of these CNVs in CHD patients indicates the rare CNVs detected in this study may contribute to AVSD pathogenesis, and the rest of them needs to be validated either by larger scale of CHD cohort study like Pediatric Cardiac Genomics Consortium (PCGC) or inventing high throughput validation experiments.

*DYRK1A, OBSCN* and *TTN* were three potential risk genes for AVSD in our cohort, whose roles in heart development has never been described in detail. *DYRK1A* in the regulation of different cellular processes involved in brain development and function (Arbones et al., 2019), autoimmunity and B Cell leukemogenesis (Li et al., 2021), and tumorigenesis (Laham et al., 2020). Joseph et al. observes that overexpressing of both *DYRK1A/DSCR1* in mice can lead vascular defects, failed heart valve development at embryonic day E13.5 (Arron et al., 2006). *OBSCN* and *TTN* are giant muscle-specific proteins, participating in a wide range of processes including myofibril assembly and maintenance, muscle protein degradation and intracellular signaling. The two proteins link to skeletal and cardiac myopathies or to muscular dystrophies (Kontrogianni-Konstantopoulos et al., 2009). We have only proposed experimental conjectures so far, further mouse models need to construct to explored the phenotypes and molecular experiments need to conduct to investigate gene function.

In conclusion, we establish a CNV architecture of AVSD patients by assembling a medium-sized case-control cohort of Chinese patients and identified a number of risky CNV loci and genes that could cause AVSD. This study advances our current understanding of molecular etiology of AVSD and promotes the genetic consults and diagnosis of AVSD now prevailing in clinics.

## Data Availability

The original contributions presented in the study are included in the article/Supplementary Material, further inquiries can be directed to the corresponding author.
