# Supplementary material for: Rare copy number variation analysis identifies disease-related variants in atrioventricular septal defect patients
Source: Front Genet. 2023 Feb 3;14:1075349. doi: 10.3389/fgene.2023.1075349 (PMC9936062; doi:10.3389/fgene.2023.1075349)
Supplement: Supplementary file 3 [file DataSheet1.PDF]

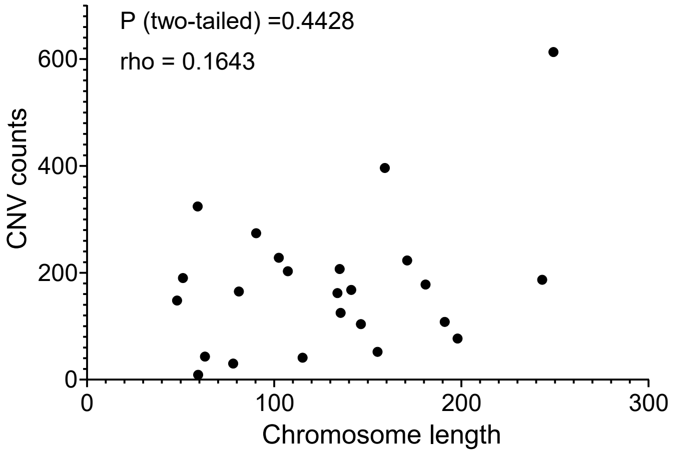

Figure S1. Nonparametric Spearman rank correlation coefficient (rho) tests between CNV counts and chromosome length. P<0.05 is considered to be significant, and P ≥ 0.05 is not statistically significant. CNV, copy number variation; rho, Spearman rank correlation coefficient
